# Sleep Characteristics and Insomnia Severity in Relation to Mediterranean Lifestyle Adherence and Psychosocial Wellbeing: Findings from the MEDIET4ALL International Survey

**DOI:** 10.3390/ejihpe16070096

**Published:** 2026-07-02

**Authors:** Achraf Ammar, Atef Salem, Khaled Trabelsi, Martha Montalvan, Bassem Bouaziz, Mohamed Ali Boujelbane, Mohamed Kerkeni, Liwa Masmoudi, Hadeel Ali Ghazzawi, Adam Tawfiq Amawi, Bekir Erhan Orhan, Raynier Zambrano-Villacres, Juliane Heydenreich, Christiana Schallhorn, Tarak Driss, Evelyn Frias-Toral, Giuseppe Grosso, Piotr Zmijewski, Haitham Jahrami, Waqar Husain, Hamdi Chtourou, Wolfgang I. Schöllhorn

**Affiliations:** 1 Department of Training and Movement Science, Institute of Sport Science, Johannes Gutenberg-University Mainz, 55122 Mainz, Germany; asalem@uni-mainz.de (A.S.); mboujelb@uni-mainz.de (M.A.B.); schoellw@uni-mainz.de (W.I.S.); 2 Research Laboratory, Molecular Bases of Human Pathology, LR19ES13, Faculty of Medicine of Sfax, University of Sfax, Sfax 3000, Tunisia; 3 Interdisciplinary Laboratory in Neurosciences, Physiology, and Psychology: Physical Activity, Health, and Learning (LINP2), UFR STAPS, Paris Nanterre University, 92000 Nanterre, France; tarak.driss@parisnanterre.fr; 4 Department of Nutrition and Food Technology, School of Agriculture, The University of Jordan, Amman 11942, Jordan; h.ghazzawi@ju.edu.jo; 5 High Institute of Sport and Physical Education of Sfax, University of Sfax, Sfax 3000, Tunisia; mohamed.kerkeni@isseps.usf.tn (M.K.); hamdi.chtourou@isseps.usf.tn (H.C.); 6 Research Laboratory: Education, Motricity, Sport and Health, EM2S, LR19JS01, High Institute of Sport and Physical Education of Sfax, University of Sfax, Sfax 3000, Tunisia; trabelsikhaled@gmail.com (K.T.); liwa.masmoudi@isseps.usf.tn (L.M.); 7 Department of Movement Sciences and Sports Training, School of Sport Science, The University of Jordan, Amman 11942, Jordan; a.amawi@ju.edu.jo; 8 School of Medicine, Universidad Católica de Santiago de Guayaquil, Guayaquil 090615, Ecuador; mmontalvanmd53@gmail.com; 9 Multimedia InfoRmation Systems and Advanced Computing Laboratory (MIRACL), University of Sfax, Sfax 3000, Tunisia; bassem.bouaziz@isims.usf.tn; 10 Higher Institute of Computer Science and Multimedia of Sfax (ISIMS), University of Sfax, Sfax 3000, Tunisia; 11 Faculty of Sports Sciences, Istanbul Aydın University, 34295 Istanbul, Türkiye; bekirerhanorhan@aydin.edu.tr; 12 Facultad de Ciencias de la Salud y Desarrollo Humano, Universidad Ecotec, Km. 13.5, Samborondón EC092302, Ecuador; razambrano@ecotec.edu.ec; 13 Department of Experimental Sports Nutrition, Faculty of Sports Sciences, Leipzig University, 04109 Leipzig, Germany; juliane.heydenreich@uni-leipzig.de; 14 Department of Sports Economics, Sociology and History, Institute of Sport Science, Johannes Gutenberg-University Mainz, 55122 Mainz, Germany; christiana.schallhorn@uni-mainz.de; 15 Escuela de Medicina, Universidad Espíritu Santo, Samborondón 0901952, Ecuador; evelynft@gmail.com; 16 Department of Biomedical and Biotechnological Sciences, University of Catania, 95123 Catania, Italy; giuseppe.grosso@unict.it; 17 Department of Biochemistry, Gdansk University of Physical Education and Sport, 80-336 Gdansk, Poland; piotr.zmijewski@insp.waw.pl; 18 Government Hospitals, Manama 323, Bahrain; hjahrami@health.gov.bh; 19 Department of Psychiatry, College of Medicine and Health Sciences, Arabian Gulf University, Manama 329, Bahrain; 20 Department of Humanities, COMSATS University Islamabad, Islamabad 45550, Pakistan; drsukoon@gmail.com; 21 Research Unit, Physical Activity, Sport, and Health, UR18JS01, National Observatory of Sport, Tunis 1003, Tunisia

**Keywords:** insomnia severity, sleep quality, Mediterranean lifestyle, life satisfaction, anxiety, social participation, multinational survey

## Abstract

Sleep is a multidimensional health domain influenced by behavioural, psychological, and lifestyle factors. However, multinational evidence integrating insomnia severity and multiple sleep outcomes within the Mediterranean lifestyle framework remains limited. This study examined correlates of insomnia severity and key sleep outcomes in adults from Mediterranean and neighbouring countries participating in the MEDIET4ALL survey. Data were collected from 4010 adults (59.5% female) across 10 countries using a standardized multilingual e-survey. Insomnia severity was assessed as primary outcome using the Insomnia Severity Index (ISI), while sleep characteristics were assessed using sleep duration, sleep latency, sleep efficiency, and subjective sleep quality. Regression analyses examined sociodemographic, health-related, Mediterranean dietary, movement-related, psychological, and social correlates. Exploratory statistical indirect association analyses involving life satisfaction were also conducted. Insomnia severity showed the highest explained variance among sleep outcomes (adjusted R^2^ ≈ 0.29). Higher insomnia severity was associated with female sex, higher body mass index, and greater depression, anxiety, and stress (β ≈ 0.15–0.17), whereas lower insomnia severity was associated with older age, better self-reported health status, higher life satisfaction, and greater adherence to Mediterranean dietary consumption patterns (β ≈ −0.04 to −0.11). Models for secondary sleep outcomes explained more modest variance and should be interpreted as exploratory. Across these outcomes, psychological well-being and distress showed the most consistent associations, while Mediterranean dietary dimensions and social participation showed smaller and outcome-specific associations. Exploratory indirect association analyses showed small but statistically significant indirect associations involving life satisfaction between Mediterranean dietary dimensions, social participation, and selected sleep outcomes, particularly sleep quality and insomnia severity. The findings confirm and contextualize established associations between sleep, psychological well-being, distress, Mediterranean lifestyle-related behaviours, and regional context within a large multinational sample. Psychological well-being and distress emerged as the most consistent correlates. Insomnia severity was the most robustly explained sleep outcome, whereas secondary sleep dimensions reflected more modest exploratory correlational profiles.

## 1. Introduction

Sleep is increasingly recognized as a fundamental pillar of health, alongside physical activity and nutrition, and plays a central role in the prevention of non-communicable diseases and the promotion of mental well-being (Watson et al., 2015; Buysse, 2014; Weng et al., 2026; Kaminsky et al., 2022). Insufficient or poor-quality sleep has been consistently associated with a wide range of adverse outcomes, including depression, anxiety, cardiometabolic disorders, impaired cognitive function, and increased mortality risk (Itani et al., 2017; Irwin, 2015; Medic et al., 2017; Jahrami et al., 2026). In this context, understanding the determinants of sleep behaviours has become a major public health priority.

Sleep is a multidimensional construct influenced by a complex interplay of biological, behavioural, psychological, and socio-environmental factors (Grandner, 2017). Growing evidence indicates that sleep outcomes such as insomnia severity, sleep quality, and sleep duration are closely linked to mental health, lifestyle behaviours, and social conditions (Baglioni et al., 2011; Grandner, 2017; Stewart et al., 2020; Yasugaki et al., 2025; Romdhani et al., 2025). In particular, psychological distress, including symptoms of anxiety, depression, and stress, has been identified as one of the strongest correlates of sleep disturbances across adult populations (Freeman et al., 2020; Moussa-Chamari et al., 2024; Jahrami et al., 2026). Conversely, positive psychological constructs such as life satisfaction and well-being appear to play a protective role, being associated with better sleep quality and lower insomnia symptoms (Shin & Kim, 2018; Willroth et al., 2022; Ogbenna et al., 2025; Radovic et al., 2026). Social participation has also emerged as an important determinant, potentially influencing sleep through psychosocial pathways related to social support and emotional regulation (Kent de Grey et al., 2018).

In parallel, lifestyle behaviours have been increasingly investigated in relation to sleep health. Dietary patterns have been associated with sleep quality and other sleep-related outcomes (Godos et al., 2021; Alghamdi & Almasaudi, 2025; Neri et al., 2025), while physical activity and sedentary behaviour have been examined within broader movement-behaviour and 24 h lifestyle frameworks relevant to sleep health (Memon et al., 2021; Kracht et al., 2024; Moussa-Chamari et al., 2024). Within this broader lifestyle-health perspective, the Mediterranean lifestyle has received particular attention as an integrative model combining healthy dietary habits, regular physical activity, adequate sleep, and strong social engagement (Sotos-Prieto et al., 2015; Diolintzi et al., 2019). Importantly, the term Mediterranean lifestyle does not imply that these behaviours occur exclusively in Mediterranean countries; rather, it refers to a historically rooted and culturally adaptable framework for studying a cluster of dietary, behavioural, and sociocultural practices that may be adopted across different populations (Sotos-Prieto et al., 2015; Diolintzi et al., 2019; Godos et al., 2024b). Recent conceptual frameworks increasingly describe Mediterranean lifestyle as a multidimensional behavioural and sociocultural construct extending beyond dietary composition alone and encompassing social participation, meal conviviality, physical activity, daily rhythm regularity, and broader lifestyle organization (Sotos-Prieto et al., 2015; Diolintzi et al., 2019; Natacci et al., 2025).

Beyond dietary composition alone, Mediterranean lifestyle adherence has been operationalized using the MEDLIFE index, which captures three complementary domains comprising Mediterranean food consumption, Mediterranean dietary habits, and broader lifestyle behaviours, including physical activity, rest, social interaction, and conviviality (Sotos-Prieto et al., 2015). This multidimensional operationalization reflects a coherent behavioural and sociocultural lifestyle pattern rather than a single dietary exposure. Consistent with this perspective, higher MEDLIFE adherence has been associated with more favourable cardiometabolic profiles, lower prevalence of metabolic syndrome, reduced cardiovascular risk factors, healthier ageing trajectories, and lower all-cause mortality in both Mediterranean and non-Mediterranean populations (Sofi et al., 2014; Mata-Fernández et al., 2021; Pavičić Žeželj et al., 2018; Hershey et al., 2021; Sotos-Prieto et al., 2021; Talavera-Rodríguez et al., 2024). These findings support the concept that Mediterranean lifestyle adherence represents a broader health-related behavioural pattern integrating dietary quality, movement behaviours, rest, and social engagement. Furthermore, adherence to Mediterranean dietary patterns has been associated with improved sleep quality and reduced insomnia symptoms (Godos et al., 2024a; Arab et al., 2025), potentially through mechanisms involving reduced inflammation and improved metabolic health (Scoditti et al., 2022; Patel & Cheung, 2025), as well as circadian and sleep-regulatory processes (Veler, 2023; St-Onge et al., 2016; Zuraikat et al., 2021). However, most studies have examined these behaviours in isolation, without accounting for their interdependence or their integration within a broader lifestyle framework (Godos et al., 2024a; Arab et al., 2025; Natacci et al., 2025).

The present study was conducted within the MEDIET4ALL project, a PRIMA-funded multinational initiative designed by an international consortium to support the transition toward a healthy, sustainable, and culturally adaptable Mediterranean lifestyle. Within this project, the MEDIET4ALL international survey was developed to assess Mediterranean lifestyle adherence and its sociodemographic, behavioural, psychosocial, and health-related correlates across Mediterranean and neighbouring countries (Ammar et al., 2025a; Ammar et al., 2026a).

Recent work from the MEDIET4ALL project has emphasized the importance of adopting a multidimensional perspective on health behaviours (Ammar et al., 2026a), showing that Mediterranean lifestyle adherence is shaped by a combination of dietary, behavioural, psychosocial, and sociodemographic determinants (Ammar et al., 2025a). This multidimensional conceptualization is also supported by recent observational evidence showing that higher adherence to the Mediterranean diet is associated with healthier lifestyle patterns and better emotional well-being in adult populations (Bühler et al., 2026). Nevertheless, the specific correlates of sleep outcomes within this integrated framework, as well as the extent to which life satisfaction may statistically link Mediterranean lifestyle-related behaviours and social participation with sleep outcomes, remain insufficiently explored, particularly in multinational populations.

Although previous systematic reviews and meta-analyses have already documented associations between Mediterranean dietary patterns and sleep outcomes, most available evidence has focused on isolated sleep indicators, particularly sleep quality, and has less frequently examined sleep within an integrated multidimensional lifestyle and psychosocial framework. Moreover, sleep is increasingly conceptualized as a multidimensional health construct comprising partially distinct but interrelated dimensions, including insomnia severity, sleep latency, sleep efficiency, sleep duration, and subjective sleep quality, each of which may reflect different behavioural, psychological, and physiological processes (Buysse, 2014; Grandner, 2017). Similarly, contemporary sleep-health frameworks emphasize that sleep is shaped not only by individual biological and behavioural factors but also by broader social, occupational, environmental, and cultural determinants (Buysse, 2014; Grandner, 2017). Consequently, examining multiple sleep dimensions may provide complementary rather than redundant information regarding the broader sleep-health profile of individuals.

Within this context, the present study primarily focused on insomnia severity as the main sleep-related outcome, while sleep duration, sleep latency, sleep efficiency, and subjective sleep quality were examined as secondary exploratory sleep dimensions. In addition to identifying correlates of these sleep outcomes, the study examined a restricted set of exploratory statistical indirect association models involving Mediterranean lifestyle-related dietary dimensions, social participation, life satisfaction, and selected sleep outcomes. These analyses were not intended to establish causal mediation, given the cross-sectional design and the strong assumptions required for causal mediation inference (Shrout, 2011; VanderWeele, 2016; Li et al., 2023). Rather, they were intended to evaluate whether life satisfaction may represent a plausible psychosocial linking construct within a broader lifestyle–well-being–sleep framework. This rationale is supported by evidence linking Mediterranean lifestyle adherence with sleep outcomes and positive psychosocial functioning (Godos et al., 2024a; Arab et al., 2025; Ammar et al., 2025a; Willroth et al., 2022), as well as evidence linking life satisfaction and social connectedness with more favourable subjective sleep profiles and lower insomnia burden (Shin & Kim, 2018; Ogbenna et al., 2025; Kent de Grey et al., 2018; Zhao et al., 2026).

Importantly, the novelty of the present study does not primarily lie in identifying entirely new Mediterranean diet–sleep associations, since several reviews and meta-analyses have already documented these relationships (Godos et al., 2024a; Arab et al., 2025). Rather, the present contribution lies in integrating multiple sleep dimensions within a broader Mediterranean lifestyle, psychosocial, and behavioural framework in a large multinational sample spanning Mediterranean and neighbouring countries, while additionally exploring whether life satisfaction statistically links selected lifestyle-related factors with sleep outcomes. Based on previous evidence, it was hypothesized that (i) psychological distress would be positively associated with poorer sleep outcomes, particularly insomnia severity, (ii) life satisfaction and social participation would be associated with more favourable sleep profiles, and (iii) Mediterranean lifestyle-related dietary dimensions and social participation would show small but statistically detectable indirect associations with selected sleep outcomes through life satisfaction.

## 2. Materials and Methods

### 2.1. Study Design and Participants

The present study used data collected within the MEDIET4ALL project, a multinational cross-sectional investigation designed to examine adherence to the Mediterranean lifestyle and its behavioural, psychosocial, and sociodemographic correlates among adults living in Mediterranean and neighbouring countries. Data were gathered using a standardized online questionnaire administered across ten participating countries: Germany, France, Italy, Spain, Luxembourg, Tunisia, Algeria, Morocco, Türkiye, and Jordan. The MEDIET4ALL survey was conducted as an international online study over a four-month period beginning in the summer of 2024. The use of a harmonized electronic survey enabled comparable data collection across diverse sociocultural settings while supporting broad participation through consortium and partner dissemination channels, including institutional webpages, mailing lists, social media platforms, and professional networks. The overall methodological framework of MEDIET4ALL, including survey development and administration procedures, has been described in detail elsewhere (Boujelbane et al., 2025a, 2025b; Ammar et al., 2025a; Ammar et al., 2025b; Ammar et al., 2026a; Ammar et al., 2026b; Ammar et al., 2026c). Briefly, the survey targeted adults aged 18 years and older residing in participating countries. Participation was voluntary and anonymous, and all respondents provided electronic informed consent before beginning the questionnaire.

The questionnaire was implemented through the secure, GDPR-compliant SoSci Survey platform hosted at Johannes Gutenberg University Mainz, Germany. To maximize accessibility across countries, the survey was made available in seven languages (English, German, French, Italian, Spanish, Arabic, and Turkish). For instruments without previously validated translations in some languages, forward–backward translation procedures were applied in accordance with established cross-cultural adaptation recommendations (Brislin, 1970). Across language versions, test–retest analyses demonstrated excellent reliability, with correlation coefficients ranging from 0.81 to 0.94 (Boujelbane et al., 2025a, 2025b; Ammar et al., 2025a; Ammar et al., 2025b; Ammar et al., 2026a; Ammar et al., 2026c). The survey initially attracted more than 8000 responses. After screening for completeness, validity, duplicate entries, internal consistency, and implausible or extreme values, approximately half were retained, yielding a final analytical dataset of 4010 responses. Before analysis, all data underwent structured quality-control procedures. These included verification of full questionnaire completion, exclusion of partial submissions, consistency checks across logically related items, identification of potential duplicate entries using IP address and timestamp information, and exclusion of implausible or extreme values. Only responses meeting predefined completeness and internal consistency criteria were included in the present analyses (Boujelbane et al., 2025a, 2025b; Ammar et al., 2025a; Ammar et al., 2025b; Ammar et al., 2026a; Ammar et al., 2026c).

The study protocol complied with the ethical principles of the Declaration of Helsinki and received approval from the Ethics Committee of the Faculty of Medicine, University of Sfax (Approval Code: 058/24). Additional details concerning recruitment procedures, survey implementation, and data privacy are available in previous MEDIET4ALL publications (Boujelbane et al., 2025a, 2025b; Ammar et al., 2025a; Ammar et al., 2025b; Ammar et al., 2026a; Ammar et al., 2026c).

### 2.2. Survey Instruments and Measured Variables

The MEDIET4ALL questionnaire was designed to assess several dimensions of the Mediterranean lifestyle together with sociodemographic characteristics, health-related behaviours, sleep, movement-related behaviours, and psychosocial indicators relevant to health and well-being.

#### 2.2.1. Sociodemographic and Lifestyle Characteristics

Participants reported age, sex, country of residence, education level, employment status, marital status, and living environment. Additional lifestyle- and health-related information included smoking status, alcohol consumption, and self-reported health status. Body weight and height were self-reported and used to calculate body mass index (BMI) (Boujelbane et al., 2025a, 2025b; Ammar et al., 2025a; Ammar et al., 2026a).

#### 2.2.2. Sleep Outcomes

The present study focused on multiple sleep-related outcomes. Sleep characteristics were assessed using selected indicators adapted from established sleep assessment tools, particularly items derived from the Pittsburgh Sleep Quality Index (PSQI), together with the Insomnia Severity Index (ISI). Four sleep dimensions were considered: sleep duration, sleep latency, sleep efficiency, and subjective sleep quality (Boujelbane et al., 2025a, 2025b; Ammar et al., 2025a; Ammar et al., 2026a; Ammar et al., 2026c).

Sleep duration was assessed as the self-reported number of hours of actual sleep obtained per night. Sleep latency was assessed as the self-reported time, in minutes, usually required to fall asleep. Sleep efficiency was calculated as the percentage ratio of actual sleep duration relative to total time spent in bed:

Sleep efficiency (%) = (actual sleep duration/total time spent in bed) × 100.

Subjective sleep quality was assessed using a four-point ordered scale ranging from “very bad” to “very good” and coded from 1 to 4, with higher scores indicating better perceived sleep quality.

Insomnia severity was assessed using the Insomnia Severity Index (ISI), a validated seven-item questionnaire covering difficulties with sleep initiation, sleep maintenance, early morning awakening, satisfaction with current sleep pattern, and the perceived daytime impact of sleep problems (Bastien et al., 2001). Each item is scored from 0 to 4, yielding a total score between 0 and 28, with higher values indicating greater insomnia severity.

#### 2.2.3. Mediterranean Lifestyle Adherence

Mediterranean lifestyle adherence was measured using the Mediterranean Lifestyle Index (MEDLIFE), a validated instrument developed to capture key features of the traditional Mediterranean lifestyle (Sotos-Prieto et al., 2015). The MEDLIFE index comprises three domains: (i) Mediterranean dietary consumption patterns, (ii) Mediterranean dietary habits, and (iii) lifestyle behaviours including physical activity, rest, social interaction, and conviviality. Items were scored according to the original MEDLIFE scoring system, with higher scores indicating stronger adherence to Mediterranean lifestyle practices (Sotos-Prieto et al., 2015; Ammar et al., 2025a).

In the present study, the two dietary domains of MEDLIFE (i.e., Mediterranean dietary consumption patterns and Mediterranean dietary habits) were examined separately because they capture related but conceptually distinct dimensions of Mediterranean lifestyle adherence. The Mediterranean dietary consumption patterns domain primarily reflects the nutritional composition and habitual intake of core Mediterranean food groups, whereas the Mediterranean dietary habits domain reflects behavioural and meal-related eating practices. This distinction was considered important to avoid conceptual overaggregation and to examine whether nutritional-compositional and behavioural-practice dimensions of Mediterranean eating showed similar or different associations with sleep outcomes.

Specifically, the Mediterranean dietary consumption patterns domain includes items related to the intake of sweets, red meat, processed meat, eggs, legumes, white meat, fish and seafood, potatoes, low-fat dairy products, nuts and olives, herbs and garnish, fruits, vegetables, olive oil, and cereals, according to the original MEDLIFE scoring system (Sotos-Prieto et al., 2015). By contrast, the Mediterranean dietary habits domain includes practices such as water or infusion intake, wine consumption in moderation, limiting salt intake, preference for whole-grain products, limiting snacks and nibbling between meals, and limiting sugar-sweetened beverages. Higher scores reflected greater adherence to Mediterranean lifestyle-related dietary behaviours.

#### 2.2.4. Physical Activity and Sedentary Behaviour

Movement-related behaviours were assessed using the short form of the International Physical Activity Questionnaire (IPAQ-SF), which estimates weekly physical activity energy expenditure in metabolic equivalent task minutes per week (MET-min/week) across walking, moderate-intensity, and vigorous-intensity activities (Craig et al., 2003). Participants also reported their average daily sitting time, which was used as an indicator of sedentary behaviour. The IPAQ-SF has been widely used in international surveillance and epidemiological studies and has demonstrated acceptable reliability and validity for population-level assessments.

#### 2.2.5. Psychological Well-Being and Distress

Life satisfaction was measured using the Short Life Satisfaction Questionnaire (SLSQ), a brief three-item instrument derived from the Satisfaction with Life Scale framework (Diener et al., 1985). Responses are scored on a 7-point Likert scale ranging from 1 (strongly disagree) to 7 (strongly agree), resulting in a total score from 3 to 21, with higher scores indicating greater life satisfaction. The SLSQ has been used in previous large-scale lifestyle and well-being studies, including earlier MEDIET4ALL analyses (Ammar et al., 2021; Boujelbane et al., 2025a, 2025b; Ammar et al., 2025a; Ammar et al., 2026a; Ammar et al., 2026b; Ammar et al., 2026c).

Psychological distress symptoms were assessed using the Depression Anxiety Stress Scales, 21-item version (DASS-21), which provides separate subscale scores for depression, anxiety, and stress (Lovibond & Lovibond, 1995). The 21 items are rated on a 4-point Likert scale from 0 (“did not apply to me at all”) to 3 (“applied to me very much or most of the time”). Higher scores indicate greater psychological distress.

#### 2.2.6. Social Participation and Technology Use

Social participation was evaluated using the Short Social Participation Questionnaire (SSPQ), which measures engagement in social activities and interpersonal interactions. The SSPQ contains 14 items, including ten Likert-type items and four dichotomous items, producing a total score ranging from 14 to 70, with higher scores reflecting greater social participation.

Technology use was assessed using the Short Technology-Use Questionnaire (STuQL), which measures the frequency of technology use in relation to daily lifestyle behaviours such as social interaction, dietary practices, and physical activity. The STuQL includes three items rated on a 5-point response scale ranging from “never” to “all the time,” with total scores from 3 to 15. Higher scores indicate more frequent technology use. Both instruments have been used in previous lifestyle-related studies and earlier MEDIET4ALL analyses (Ammar et al., 2021; Boujelbane et al., 2025a, 2025b; Ammar et al., 2025a; Ammar et al., 2025b; Ammar et al., 2026a; Ammar et al., 2026c).

### 2.3. Statistical Analysis

Most statistical analyses were conducted using SPSS version 29 (IBM Corp., Chicago, IL, USA). Additional sensitivity and supplementary analyses, including the sleep-outcome correlation matrix, the interaction sensitivity analysis, and the ordinal logistic regression sensitivity analysis, were conducted using R version 4.5.1 statistical software (R Foundation for Statistical Computing, Vienna, Austria). Descriptive statistics were used to summarize the characteristics of the sample and the distribution of study variables. Continuous variables are presented as means and standard deviations, whereas categorical variables are reported as frequencies and percentages. To examine the degree of overlap among sleep outcomes, Spearman rank-order correlations were calculated among ISI total score, sleep duration, sleep latency, sleep efficiency, and subjective sleep quality. These correlations are reported in the Supplementary Materials to clarify whether the sleep outcomes represented overlapping or partially distinct sleep dimensions.

To examine associations between sleep outcomes and conceptually grouped domains of variables within the broader MEDIET4ALL framework, hierarchical multiple linear regression analyses were conducted. Insomnia severity, assessed using the ISI total score, was considered the primary sleep-related outcome because the ISI captures multiple dimensions of insomnia symptom burden, including sleep initiation, sleep maintenance, early morning awakening, dissatisfaction with sleep, perceived distress, interference, and daytime impact (Bastien et al., 2001). Sleep duration, sleep latency, sleep efficiency, and subjective sleep quality were analysed as secondary exploratory sleep outcomes. Variables were entered sequentially in blocks representing sociodemographic characteristics, health-related factors, Mediterranean lifestyle-related dietary dimensions, movement-related variables, psychological variables, and social/technology-related factors. The hierarchical sequence was theory-informed and intended primarily to examine the incremental variance explained by conceptually grouped domains rather than to infer causal ordering or isolated causal effects. Sociodemographic and health-related variables were entered first because they represent relatively stable background characteristics and general health-related covariates within the MEDIET4ALL conceptual framework. Behavioural and psychosocial domains were subsequently added to evaluate whether associations persisted after progressive adjustment across broader lifestyle-related and psychological domains. Importantly, the final fully adjusted and parsimonious regression models were considered the principal inferential models of the study and are emphasized throughout the manuscript, whereas the hierarchical block sequence was used primarily to describe progressive variance partitioning across conceptual domains within the MEDIET4ALL framework rather than infer isolated causal effects of individual variables. The final parsimonious model retained the variables independently associated with insomnia severity after full adjustment across the examined domains. An additional sensitivity analysis was conducted to examine whether the association between MEDLIFE dietary consumption patterns and insomnia severity differed according to region. For this purpose, an interaction term between region and centred MEDLIFE dietary consumption patterns was added to the final ISI regression model. Because subjective sleep quality was measured using a four-level ordinal scale, a supplementary ordinal logistic regression sensitivity analysis was conducted to evaluate the robustness of the findings obtained from the primary linear regression analyses.

In addition, exploratory statistical indirect association analyses were conducted to examine whether life satisfaction statistically linked selected Mediterranean lifestyle-related dietary dimensions and social participation variables with sleep outcomes. The retained models examined indirect associations between MEDLIFE dietary dimensions and insomnia severity or subjective sleep quality through life satisfaction, as well as indirect associations between social participation and subjective sleep quality through life satisfaction.

The retained models were restricted to theoretically plausible pathways supported by the final regression models and by previous literature linking Mediterranean lifestyle adherence with psychosocial well-being and sleep-related health (Godos et al., 2024a; Arab et al., 2025; Ammar et al., 2026b). In particular, life satisfaction was selected as the primary linking psychosocial construct because positive well-being indicators have repeatedly been associated with both healthier lifestyle behaviours and more favourable sleep profiles (Willroth et al., 2022; Ogbenna et al., 2025).

Given the cross-sectional design, these analyses were interpreted cautiously as exploratory statistical indirect association models and were not used to infer temporal ordering or causal mechanisms (Shrout, 2011; VanderWeele, 2016; Li et al., 2023). All retained models were adjusted for age, sex, region, education level, employment status, BMI, smoking status, alcohol consumption, and self-reported health status.

Indirect associations were estimated using a bootstrapping procedure with 5000 resamples and corresponding 95% confidence intervals. An indirect effect was considered statistically significant when the bootstrapped 95% confidence interval did not include zero (Preacher & Hayes, 2008; Hayes, 2018). Effect sizes were interpreted conservatively, with emphasis placed on direction, uncertainty, and practical magnitude rather than mechanistic interpretation.

Prior to model interpretation, standard regression assumptions, including linearity, normality of residuals, homoscedasticity, and multicollinearity, were examined. Multicollinearity was assessed using variance inflation factors (VIF) and tolerance statistics, and the corresponding diagnostics for the fully adjusted ISI model are presented as Supplementary Materials. Tolerance values >0.20 and VIF values <5 were considered indicative of no problematic multicollinearity (Hair et al., 2010). All statistical tests were two-tailed, and statistical significance was set at *p* < 0.05.

## 3. Results

### 3.1. Descriptive Characteristics of the Study Population

The analytical sample comprised 4010 adults from ten Mediterranean and neighbouring countries. A comprehensive description of participant characteristics has been reported elsewhere (Boujelbane et al., 2025a, 2025b; Ammar et al., 2026a; Ammar et al., 2026b; Ammar et al., 2026c) and is briefly summarized here for contextual purposes. The sample included both sexes (59.5% female) and reflected diverse educational (58.8% holding high education diploma), occupational (50.7% employed), and residential backgrounds (66.3% residing in urban areas). Substantial variability was observed in Mediterranean lifestyle adherence, with 31.1%, 46.8%, and 22.1% of participants classified as having low, medium, and high adherence, respectively. Similarly, heterogeneity was evident in movement behaviours, with 60.3%, 20.2%, and 19.5% of participants categorized as having low, medium, and high physical activity levels, respectively, and a mean sitting time of 5.59 ± 3.04 h per day. Social participation levels were also heterogeneous, with most participants classified as “sometimes socially active” (50.7%), followed by “often socially active” (26.9%), and a mean SSPQ score of 37.90 ± 10.06. Regarding sleep characteristics, mean sleep duration was 6.82 ± 1.70 h/night, mean sleep latency was 30.41 ± 33.69 min, mean sleep efficiency was 93.14 ± 7.15%, mean subjective sleep quality score was 2.82 ± 0.79, and mean ISI score was 9.66 ± 5.82. Overall, 43.1% of participants reported sleep duration below age-specific recommendations, 41.8% reported sleep latency >20 min, and 11.0% had sleep efficiency <85%. Regarding insomnia severity, 38.4% of participants were classified as having no insomnia, 40.3% subthreshold insomnia, 18.5% moderate insomnia, and 2.9% severe insomnia.

### 3.2. Correlations Among Sleep Outcomes

Spearman correlations among sleep outcomes are presented in Supplementary Table S1. The sleep variables were significantly interrelated but not redundant, supporting their interpretation as partially distinct dimensions of sleep health. As expected, higher insomnia severity was associated with longer sleep latency, lower sleep efficiency, poorer subjective sleep quality, and shorter sleep duration.

### 3.3. Hierarchical Regression Analyses of Insomnia Severity (ISI)

Hierarchical multiple linear regression analyses examining insomnia severity (ISI) correlates, the primary sleep-related outcome of the present study, are presented in Supplementary Table S2, while the final fully adjusted parsimonious model is presented in Table 1. Among all sleep outcomes examined, insomnia severity demonstrated the highest explained variance and the most robust explanatory profile. Across the sequential models, the proportion of explained variance increased progressively from 3.4% in Model 1 to 29.7% in Model 6 (Table S2), with the largest incremental improvement observed after the inclusion of psychological variables (ΔR^2^ = 0.202), followed by the addition of health-related variables (ΔR^2^ = 0.053). By contrast, Mediterranean dietary variables and movement-related variables contributed comparatively smaller incremental increases in explained variance (ΔR^2^ = 0.001 to 0.006).

In the final parsimonious model (explained variance = 29.4%), higher insomnia severity was positively associated after adjustment with female sex (B = 0.619, 95% CI: 0.298 to 0.940), higher body mass index (B = 0.036, 95% CI: 0.003 to 0.070), depressive symptoms (B = 0.170, 95% CI: 0.114 to 0.226), anxiety symptoms (B = 0.208, 95% CI: 0.154 to 0.262), and stress symptoms (B = 0.199, 95% CI: 0.142 to 0.256). Conversely, lower insomnia severity was associated after adjustment with older age (B = −0.028, 95% CI: −0.039 to −0.017), residence in Mediterranean compared with non-Mediterranean countries (B = −0.761, 95% CI: −1.090 to −0.431), better self-reported health status (B = −0.913, 95% CI: −1.187 to −0.639), greater adherence to Mediterranean dietary consumption patterns (B = −0.113, 95% CI: −0.192 to −0.034), higher life satisfaction (B = −0.140, 95% CI: −0.177 to −0.103), and lower smoking behaviour (B = −0.243, 95% CI: −0.435 to −0.051).

Among the retained variables, anxiety showed the strongest positive standardized association with insomnia severity (β = 0.169), followed by stress (β = 0.163) and depression (β = 0.146), whereas life satisfaction showed the strongest negative standardized association (β = −0.109), followed by self-reported health status (β = −0.094), age (β = −0.074), and Mediterranean dietary consumption patterns (β = −0.038). Variables such as education, employment status, marital status, living environment, alcohol consumption, Mediterranean dietary habits, physical activity, sitting time, social participation, and technology use were not retained in the final model.

Collinearity diagnostics for the fully adjusted ISI model are presented in Supplementary Table S3. Tolerance values ranged from 0.288 to 0.990, and VIF values ranged from 1.010 to 3.468, indicating no problematic multicollinearity among the included variables. The additional sensitivity analysis revealed a statistically significant interaction between region and MEDLIFE dietary consumption patterns in relation to insomnia severity (B = 0.672, β = 0.034, *p* = 0.041; Supplementary Table S4). This indicates that the inverse association between Mediterranean dietary consumption patterns and insomnia severity differed according to region and appeared stronger in non-Mediterranean than in Mediterranean regions.

### 3.4. Final Regression Models of Sleep Outcomes

The final parsimonious regression models examining secondary exploratory sleep outcomes are presented in Table 2. Compared with insomnia severity, these models explained substantially smaller proportions of variance, with adjusted R^2^ values ranging from 0.046 for sleep duration to 0.117 for subjective sleep quality.

Across these secondary exploratory outcomes, several statistically consistent but modest associations were observed. Psychological and well-being-related variables showed the most consistent associations within these modestly explained models. In particular, higher life satisfaction was positively associated with sleep efficiency (β = 0.121, *p* < 0.001) and sleep quality (β = 0.171, *p* < 0.001), and negatively associated with sleep latency (β = −0.109, *p* < 0.001), indicating shorter time to fall asleep. Anxiety was positively associated with sleep duration (β = 0.114, *p* < 0.001) and showed the strongest association with sleep latency (positive: β = 0.24, *p* < 0.001) and sleep efficiency (negative: β = 0.22, *p* < 0.001), while stress was negatively associated with sleep duration (β = −0.147, *p* < 0.001) and sleep quality (β = −0.162, *p* < 0.001). Social participation (SSPQ) was positively associated with sleep duration (β = 0.059, *p* < 0.001) and sleep quality (β = 0.082, *p* < 0.001).

Sociodemographic and health-related variables also showed outcome-specific associations. Older age was associated with shorter sleep duration (β = −0.099, *p* < 0.001), while female sex was associated with longer sleep latency (β = 0.063, *p* < 0.001) and lower sleep efficiency and quality (β = −0.02 to −0.062, *p* < 0.001). Better self-reported health status was consistently associated with more favourable sleep outcomes, including longer sleep duration (β = 0.054, *p* = 0.001), higher sleep efficiency (β = 0.043, *p* = 0.005), and better sleep quality (β = 0.106, *p* < 0.001). Smoking and alcohol consumption were positively associated with longer sleep latency (β = 0.033, *p* = 0.033 and β = 0.038, *p* = 0.016, respectively), while unstable employment status was associated with higher sleep latency (β = 0.053, *p* < 0.001) and lower sleep efficiency (β = −0.04, *p* = 0.005).

Regarding Mediterranean lifestyle variables, the observed associations were small and outcome-specific. Both MEDLIFE dietary consumption patterns and dietary habits were inversely associated with sleep latency (β = −0.034, *p* = 0.029 and β = −0.033, *p* = 0.031, respectively), indicating shorter time to fall asleep among individuals with higher adherence. Both dimensions were also positively associated with sleep quality (β = 0.038, *p* = 0.012 and β = 0.031, *p* = 0.045, respectively).

Movement-related behaviours showed outcome-specific associations. Higher physical activity (IPAQ score) was positively associated with sleep latency (β = 0.049, *p* = 0.001) and negatively associated with sleep efficiency and quality (β = −0.043 to −0.047, *p* = 0.002 to 0.005), while greater sitting time was positively associated with longer sleep latency (β = 0.036, *p* = 0.017).

Additional ordinal logistic regression sensitivity analyses conducted for subjective sleep quality yielded patterns broadly consistent with the primary linear regression analyses. The ordinal model showed significant improvement over the null model, χ^2^(35) = 568.73, *p* < 0.001, with Nagelkerke pseudo-R^2^ = 0.147. Higher MEDLIFE score, life satisfaction, perceived health status, and social participation were associated with greater odds of reporting better sleep quality, whereas higher stress and residence in non-Mediterranean regions were associated with poorer sleep quality. However, because the proportional odds assumption was statistically violated, these findings were interpreted cautiously as sensitivity analyses (Supplementary Table S5).

### 3.5. Exploratory Statistical Indirect Association Analyses Involving Life Satisfaction

**Figure 1:**
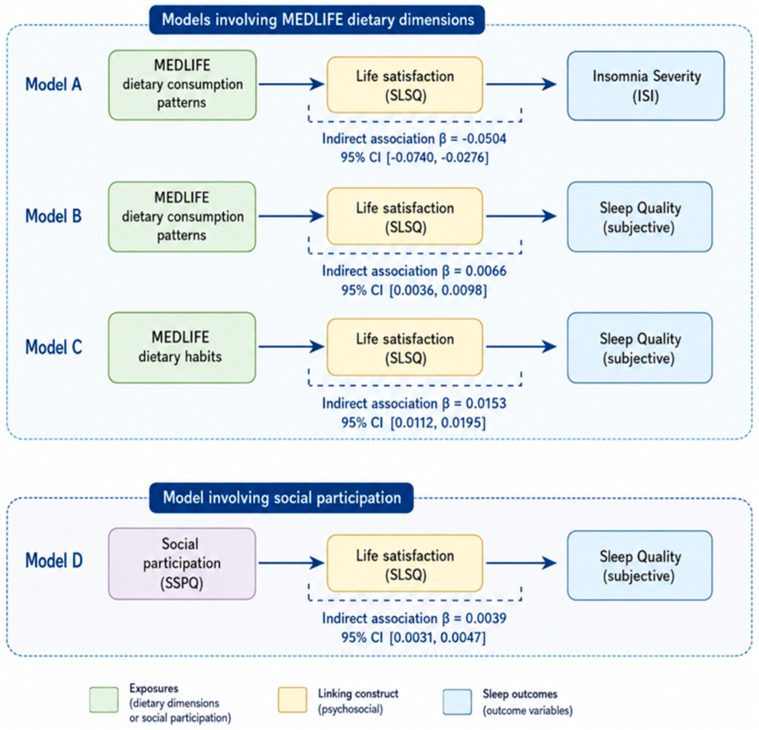
Exploratory statistical indirect association models. All models are adjusted for age, sex, region, education level, employment status, BMI, smoking status, alcohol consumption, and self reported health status. β = Standardized indirect association (bootstrapped estimate); CI = Confidence interval (bias-corrected 95% boostrap). Note: These models represent statistical indirect associations only and do not imply temporal ordering or causal mechanisms.

The retained exploratory statistical indirect association models are illustrated in Figure 1, while detailed numerical results are provided in Supplementary Table S6.

A small but statistically significant indirect association was observed between MEDLIFE dietary consumption patterns and insomnia severity through life satisfaction (indirect β = −0.0504, 95% CI: −0.0740 to −0.0276). Small indirect associations through life satisfaction were also observed between MEDLIFE dietary consumption patterns and subjective sleep quality (indirect β = 0.0066, 95% CI: 0.0036 to 0.0098), as well as between MEDLIFE dietary habits and subjective sleep quality (indirect β = 0.0153, 95% CI: 0.0112 to 0.0195). Comparing the two Mediterranean dietary dimensions, the indirect association was numerically larger for Mediterranean dietary habits than for dietary consumption patterns in relation to subjective sleep quality, although both indirect associations were statistically significant.

In addition, a small but statistically significant indirect association through life satisfaction was observed between social participation and subjective sleep quality (indirect β = 0.0039, 95% CI: 0.0031 to 0.0047).

## 4. Discussion

The present study examined insomnia severity together with several secondary exploratory sleep dimensions within the MEDIET4ALL framework and showed that sleep outcomes were associated with a multidimensional set of psychological, lifestyle-related, health-related, psychosocial, and sociodemographic factors. Overall, insomnia severity emerged as the most robust and best-explained sleep-related outcome, with psychological variables accounting for the largest proportion of explained variance. In particular, anxiety, stress, depression, and lower life satisfaction showed the strongest and most consistent associations with greater insomnia severity. Across the remaining sleep outcomes, higher life satisfaction was consistently associated with more favourable sleep profiles, including better sleep efficiency and subjective sleep quality and shorter sleep latency, whereas psychological distress variables demonstrated differential adverse associations with sleep duration, latency, efficiency, and quality.

Mediterranean lifestyle-related dietary dimensions and social participation showed comparatively smaller and more outcome-specific associations with sleep outcomes, particularly shorter sleep latency and better subjective sleep quality. In the exploratory indirect association analyses, life satisfaction emerged as a relevant psychosocial variable statistically linking Mediterranean dietary dimensions and social participation with selected sleep outcomes, whereas the anxiety-based model was not retained in the revised analyses. However, because of the cross-sectional design, these indirect associations should be interpreted cautiously as hypothesis-generating statistical relationship patterns rather than evidence of causal or mechanistic pathways.

Importantly, the models for sleep duration, sleep latency, sleep efficiency, and subjective sleep quality explained substantially smaller proportions of variance than the insomnia severity models, suggesting that the current correlate set captured only part of the variability in these secondary sleep dimensions and that additional biological, occupational, environmental, contextual, and chronobiological determinants not assessed in the present survey likely contribute importantly to these outcomes. These findings therefore support the view that different sleep dimensions may reflect overlapping but partially distinct behavioural, psychological, and physiological processes rather than interchangeable manifestations of a single sleep construct.

Overall, the present findings largely align with already established literature linking psychological distress, life satisfaction, Mediterranean lifestyle-related behaviours, and sleep outcomes. Accordingly, the principal contribution of the present study does not primarily reside in identifying entirely novel associations, but rather in integrating multiple sleep dimensions within a broader multinational Mediterranean lifestyle and psychosocial framework spanning Mediterranean and neighbouring countries.

### 4.1. Psychological Well-Being, Psychological Distress, and Sleep Outcomes

A central finding of the present study is the strong and consistent role of psychological well-being and psychological distress across sleep outcomes. In particular, insomnia severity was most strongly associated with anxiety, stress, depression, and lower life satisfaction, while higher life satisfaction was also linked to shorter sleep latency, greater sleep efficiency, and better sleep quality. These results are fully consistent with the broader literature indicating that sleep and mental health are tightly interconnected and likely bidirectional (Yasugaki et al., 2025; Stewart et al., 2020; Jahrami et al., 2026; Romdhani et al., 2025). This bidirectional relationship has been extensively documented across epidemiological, clinical, and experimental research, suggesting that sleep disturbances can both contribute to and result from psychological distress through shared neurobiological and behavioural pathways (Freeman et al., 2020; Irwin, 2015). Longitudinal and meta-analytic evidence shows that insomnia is not only highly comorbid with depression and anxiety but also predicts later mental health problems, while improvements in sleep are associated with reductions in depression, anxiety, and stress symptoms (Hertenstein et al., 2019; Scott et al., 2021; Firth et al., 2020). More broadly, chronic sleep disruption has been linked to adverse short- and long-term mental and physical health outcomes, further reinforcing the central role of sleep within overall health frameworks (Medic et al., 2017; Grandner, 2017). Recent reviews have continued to emphasize that insomnia and broader sleep disturbance are closely intertwined with common mental health problems across adulthood (Palagini et al., 2024; Whitehead & Horton, 2025).

The present findings are consistent with and extend this evidence by showing that positive psychological functioning, not only distress, was also relevant to sleep within the MEDIET4ALL framework. Life satisfaction emerged as the strongest negative standardized correlate of insomnia severity and one of the strongest positive correlates of sleep quality and sleep efficiency. This is in line with recent population-based evidence suggesting that greater life satisfaction is associated with better sleep health, including more restorative sleep and fewer insomnia symptoms (Ogbenna et al., 2025). Additional evidence indicates that good sleep quality may itself act as a protective or “buffering” factor for well-being, attenuating the impact of daily affective fluctuations on global life satisfaction (Willroth et al., 2022), while positive sleep experiences have been linked to more favourable cognitive and emotional appraisals of life circumstances (Shin & Kim, 2018). It is also concordant with other work showing that sleep quality, mental health, and broader well-being are closely interrelated across diverse populations (Moussa-Chamari et al., 2024; Romdhani et al., 2025; Jahrami et al., 2026). Together, these findings reinforce the view that sleep should not be framed solely as the absence of disturbance, but also as a behavioural correlate of positive well-being and quality of life.

A further notable finding was that the role of psychological variables differed across sleep dimensions. Anxiety showed the strongest positive association with sleep latency and was also related to longer sleep duration, whereas stress was more strongly linked to shorter sleep duration and poorer sleep quality. This pattern suggests that different dimensions of psychological distress may map onto different dimensions of sleep. Such heterogeneity is plausible, as anxiety is often linked to hyperarousal and difficulty initiating sleep, whereas stress may be more broadly related to poor sleep continuity, reduced restorative sleep, and lower perceived sleep quality (Palagini et al., 2024; Zhang et al., 2024). From a mechanistic perspective, anxiety-related hyperactivation of cognitive and physiological arousal systems may prolong sleep onset, whereas stress-related dysregulation of neuroendocrine and inflammatory pathways may impair sleep maintenance and subjective recovery (Irwin, 2015; Freeman et al., 2020). These distinctions are important because they support the analytical strategy adopted in the present paper, namely examining multiple sleep outcomes separately rather than reducing sleep to a single composite indicator.

Within the broader MEDIET4ALL framework, these findings also complement recent analyses, in which insomnia severity and subjective sleep quality emerged as central correlates of psychological well-being and distress (Ammar et al., 2026b). Taken together, the current and previous MEDIET4ALL findings suggest that sleep is closely interconnected with psychological functioning and broader lifestyle-related factors within this multinational dataset. Rather than representing an isolated behavioural domain, sleep appears to occupy an important position within a broader lifestyle–mental health network involving psychosocial well-being, Mediterranean lifestyle-related behaviours, and health-related factors. However, because of the cross-sectional design, these relationships should be interpreted cautiously as interrelated correlational patterns rather than directional or mechanistic pathways.

An important implication of these findings is that different sleep dimensions demonstrated partially distinct correlational profiles despite being interrelated. This supports contemporary multidimensional conceptualizations of sleep health (Buysse, 2014), which emphasize that insomnia severity, sleep duration, sleep efficiency, sleep latency, and subjective sleep quality should not necessarily be treated as interchangeable indicators. Instead, these dimensions may capture overlapping but partially distinct aspects of behavioural, psychological, and physiological functioning. The present findings therefore support the analytical decision to examine multiple sleep dimensions separately while simultaneously recognizing insomnia severity as the most robust and best-explained primary sleep outcome within the current dataset. Together with the supplementary correlation and collinearity analyses, these findings support the interpretation that the examined sleep outcomes and explanatory domains were interrelated but not redundant, and that the regression models should therefore be interpreted as adjusted correlational profiles rather than isolated predictive effects.

### 4.2. Mediterranean Lifestyle, Social Participation, and Indirect Association Patterns with Sleep

The present findings also provide additional support for previously documented associations between Mediterranean lifestyle-related dietary behaviours and sleep outcomes. By analysing the two dietary MEDLIFE domains separately, this paper shows that Mediterranean dietary consumption patterns were associated with lower insomnia severity and shorter sleep latency, while both Mediterranean dietary consumption patterns and dietary habits were positively associated with sleep quality. This distinction is informative because the two domains capture related but different components of lifestyle adherence. Mediterranean dietary consumption patterns primarily reflect the nutritional composition and frequency of consumption of core Mediterranean food groups, whereas Mediterranean dietary habits reflect behavioural and meal-related eating practices. These two domains are consistent with the broader conceptualization of the Mediterranean lifestyle as a multidimensional behavioural model in which dietary quality, eating practices, movement, rest, and social interaction interact rather than operate in isolation (Sotos-Prieto et al., 2015; Diolintzi et al., 2019). The present findings therefore suggest that both the nutritional profile of the Mediterranean lifestyle and the behavioural quality of eating practices may be relevant to sleep, although not necessarily through identical pathways. This interpretation is consistent with previous MEDIET4ALL work showing that Mediterranean lifestyle adherence is shaped by a multidimensional combination of demographic, behavioural, and psychosocial determinants rather than by dietary intake alone.

These results are broadly consistent with the recent sleep–nutrition literature. A 2024 systematic review concluded that stronger adherence to the Mediterranean diet is generally associated with better sleep quality and more favourable sleep features (Godos et al., 2024a), and a more recent systematic review and meta-analysis similarly linked the Mediterranean diet with sleep duration, sleep quality, chronotype, and lower insomnia burden (Arab et al., 2025). A further systematic review of observational studies also supported a positive relationship between Mediterranean diet adherence and sleep pattern, although it emphasized heterogeneity across populations and measures (Fallah et al., 2024). Earlier reviews had already shown that healthier dietary profiles, including Mediterranean-style patterns, tend to cluster with better sleep quality, thereby providing a broader nutritional context for the present findings (Godos et al., 2021; Scoditti et al., 2022). More recent evidence from adult populations also supports a direct association between healthier dietary habits and better sleep quality, even outside the Mediterranean region, suggesting that the diet–sleep link may reflect both specific dietary composition and broader behavioural regularity (Alghamdi & Almasaudi, 2025; Patel & Cheung, 2025). The present findings are broadly consistent with this literature and provide a multinational, integrative extension by examining these associations within a broader psychosocial framework while simultaneously accounting for multiple behavioural and psychological correlates. Specifically, they showed within one multinational sample, that Mediterranean dietary dimensions remain associated with sleep outcomes even after adjustment for psychological, movement-related, and sociodemographic covariates. They also suggest that the specific sleep outcomes most closely related to Mediterranean dietary dimensions may be sleep latency, sleep quality, and insomnia severity rather than sleep duration alone. One plausible explanation is that Mediterranean dietary patterns may influence sleep through multiple pathways, including reduced inflammatory burden, improved metabolic regulation, and better circadian alignment, all of which have been implicated in sleep regulation (Scoditti et al., 2022; Veler, 2023). However, these dietary associations were small and were observed primarily within secondary sleep models with modest explained variance; therefore, they should be interpreted as correlational signals consistent with previous diet–sleep literature rather than evidence that Mediterranean dietary behaviours are major determinants of secondary sleep outcomes.

A particularly noteworthy finding was that region remained independently associated with insomnia severity after adjustment for dietary, psychological, health-related, and behavioural variables, with a standardized association larger than that observed for MEDLIFE dietary consumption patterns. This suggests that broader regional and sociocultural context may capture dimensions of Mediterranean lifestyle not fully represented by the measured MEDLIFE dietary domains.

Contemporary conceptualizations of the Mediterranean lifestyle emphasize that it should not be reduced to dietary composition alone, but understood as a multidimensional behavioural and sociocultural model involving meal timing and conviviality, physical activity, social participation, daily rhythm regularity, environmental context, and broader cultural organization of daily life (Sotos-Prieto et al., 2015; Diolintzi et al., 2019). Accordingly, the independent regional association observed here may reflect broader contextual characteristics such as social rhythm regularity, culturally embedded meal patterns, family cohesion, sleep timing norms, work schedules, climate, urbanization, and environmental stressors influencing sleep health beyond the specific dietary variables assessed.

This interpretation is consistent with multidimensional sleep-health frameworks proposing that sleep outcomes are shaped not only by individual behaviours but also by broader social, occupational, environmental, and cultural conditions (Buysse, 2014; Grandner, 2017). Recent global sleep-health perspectives similarly emphasize that sleep duration, sleep quality, and sleep consistency are shaped by broader social, occupational, environmental, and cultural determinants beyond individual lifestyle behaviours alone (DelRosso, 2025). In multinational populations, region may therefore operate as a proxy indicator for contextual and behavioural exposures that are difficult to fully capture through individual-level dietary or psychosocial variables.

Importantly, the additional interaction analysis revealed a statistically significant moderation effect of region on the association between Mediterranean dietary consumption patterns and insomnia severity. Specifically, the inverse association between Mediterranean dietary consumption patterns and insomnia severity appeared stronger in non-Mediterranean than in Mediterranean regions. Although the magnitude of this interaction was relatively small, it nevertheless suggests that broader contextual and sociocultural environments may influence how Mediterranean lifestyle-related dietary behaviours relate to sleep-health profiles across populations.

The exploratory indirect association analyses provide a cautious statistical extension of these findings. Life satisfaction statistically linked Mediterranean dietary consumption patterns with insomnia severity and linked both Mediterranean dietary dimensions with subjective sleep quality. In addition, life satisfaction statistically linked social participation with subjective sleep quality.

These patterns are conceptually plausible within an integrated lifestyle–well-being–sleep framework. Recent systematic reviews and meta-analyses suggest that stronger adherence to Mediterranean dietary patterns is associated with better sleep quality and lower insomnia burden (Godos et al., 2024a; Arab et al., 2025), while parallel evidence indicates that healthier lifestyle behaviours are also associated with more favourable psychosocial functioning and higher subjective well-being (Willroth et al., 2022; Ogbenna et al., 2025). In addition, social participation and social connectedness have been linked to both life satisfaction and better sleep outcomes across adult populations (Kent de Grey et al., 2018; Seo & Mattos, 2024). Accordingly, the observed indirect associations are consistent with the possibility that positive psychosocial functioning may represent one component of the broader relationship between Mediterranean lifestyle adherence and sleep health. This interpretation is also congruent with broader MEDIET4ALL findings, in which life satisfaction emerged as a central correlate of more favourable psychological and sleep-related profiles across multiple analyses (Ammar et al., 2026b). Taken together, these findings support the possibility that positive psychosocial functioning may represent one relevant psychosocial component within the broader Mediterranean lifestyle–sleep relationship.

However, these analyses do not establish that Mediterranean dietary behaviours or social participation improve sleep through life satisfaction. As emphasized in methodological literature on cross-sectional mediation analyses (Shrout, 2011; VanderWeele, 2016; Li et al., 2023), statistical indirect associations observed in cross-sectional data cannot establish temporal ordering or causal mechanisms and may reflect bidirectional relationships, shared variance, or residual confounding. The present findings should therefore be interpreted as hypothesis-generating rather than mechanistic evidence.

The relatively small magnitude of the indirect associations is also important. Although statistically significant in this large multinational sample, these effects should not be overstated as clinically meaningful mechanisms. Rather, they suggest that positive psychosocial well-being may represent a relevant correlate within the broader Mediterranean lifestyle–sleep relationship. Longitudinal and intervention-based studies using repeated measures and objective sleep assessments will be necessary to determine whether changes in Mediterranean lifestyle adherence or social participation precede subsequent changes in life satisfaction and sleep-related outcomes.

Social participation also deserves particular emphasis. Although SSPQ was not retained as a direct correlate of insomnia severity, it was positively associated with sleep duration and sleep quality, and life satisfaction statistically linked social participation with subjective sleep quality. This is consistent with broader research indicating that social connectedness and social support are positively associated with sleep quality and may buffer the adverse impact of psychological strain on sleep (Kent de Grey et al., 2018; Seo & Mattos, 2024). The meta-analysis by Kent de Grey et al. (2018) further suggests that social support may benefit sleep across different populations and sleep indicators, supporting the idea that sleep health is partly socially embedded rather than purely individual. Within the MEDIET4ALL framework, this is especially relevant because previous regional and cross-country analyses have shown that social participation is one of the most variable lifestyle dimensions across contexts and may represent an important environmental and cultural resource shaping multiple health behaviours (Boujelbane et al., 2025b; Ammar et al., 2026a; Ammar et al., 2026c). The current findings therefore suggest that sleep quality may represent one domain statistically interconnected with the psychosocial dimension of Mediterranean lifestyle within the broader lifestyle–well-being framework. This interpretation also fits with emerging 24 h movement and lifestyle frameworks, which emphasize that health outcomes are shaped by interacting behavioural domains across the full day rather than by isolated exposures considered separately (Kracht et al., 2024).

Taken together, these findings provide an integrative and contextualized extension of existing diet–sleep and psychosocial–sleep evidence. Their added value lies in examining established sleep-related correlates within one multinational Mediterranean lifestyle framework, distinguishing dietary consumption patterns from dietary habits, comparing associations across multiple sleep dimensions, and highlighting the relevance of broader regional and psychosocial context.

From a public health perspective, the present findings support an integrated conceptualization of sleep within broader Mediterranean lifestyle and psychosocial-health frameworks. This is consistent with the broader idea that Mediterranean lifestyle adherence reflects a constellation of mutually reinforcing behaviours rather than a single nutritional pattern. The observed associations also highlight the importance of considering the interaction between dietary behaviours, psychosocial well-being, and social participation when examining sleep health in real-world settings. However, intervention and longitudinal studies are needed before concluding that modifying Mediterranean lifestyle-related behaviours, psychosocial well-being, or social participation directly improves sleep outcomes.

### 4.3. Sociodemographic, Health-Related, and Movement-Behaviour Correlates of Sleep

Several sociodemographic and health-related variables also showed meaningful associations with sleep outcomes. Better self-reported health status was consistently linked with lower insomnia severity, longer sleep duration, higher sleep efficiency, and better sleep quality. This is in line with the broader MEDIET4ALL programme, where self-reported health status was closely tied to multiple lifestyle, movement, and psychosocial variables, and where sedentary behaviour, distress, and lifestyle adherence were all relevant to health status profiles. More broadly, subjective health perception has been identified as a robust integrative indicator of overall physiological and psychosocial functioning, closely associated with sleep health and general well-being across populations (Grandner, 2017; Medic et al., 2017). In the present paper, the consistency of the health-status associations across several sleep outcomes reinforces the view that subjective health perception is not merely a parallel correlate, but a meaningful marker of the broader physiological and psychosocial context in which sleep occurs.

Age and sex were also relevant. Older age was associated with lower insomnia severity but shorter sleep duration, while female sex was associated with higher insomnia severity, longer sleep latency, and poorer sleep efficiency and sleep quality. This pattern is broadly coherent with the epidemiology of sleep across adulthood. Large-scale epidemiological studies have shown that ageing is typically accompanied by reductions in total sleep time and changes in sleep architecture, including lighter and more fragmented sleep, whereas insomnia complaints are often more strongly reported in younger and middle-aged adults facing higher psychosocial demands (Medic et al., 2017; Grandner, 2017). Likewise, women generally report a higher burden of insomnia and poorer subjective sleep quality than men, despite complex sex differences across specific sleep dimensions (Howarth & Miller, 2024; Spytska, 2024). These sex differences have been partly attributed to hormonal, psychosocial, and behavioural factors, including stress reactivity and caregiving roles, which may disproportionately affect sleep regulation (Freeman et al., 2020). These results suggest that sleep outcomes should not be discussed as a unitary construct, because age and sex may relate differently to insomnia symptoms, quantity, efficiency, and perceived quality.

Smoking and alcohol were mainly related to sleep latency, and smoking was also linked to insomnia severity. These findings are plausible given growing evidence that smoking disrupts sleep architecture and undermines sleep quality, while alcohol may initially shorten sleep onset latency but often worsens later-night sleep continuity and overall sleep quality (Grigoriou et al., 2024; Nakajima et al., 2025; Chaput, 2026). Experimental and epidemiological studies further indicate that nicotine acts as a stimulant affecting central nervous system arousal, while alcohol disrupts rapid eye movement (REM) sleep and increases sleep fragmentation, leading to poorer overall sleep quality despite perceived short-term sedative effects (Medic et al., 2017; Irwin, 2015). In the present study, both smoking and alcohol were associated with longer sleep latency rather than shorter latency, which may reflect habitual use patterns, tolerance, or other unmeasured behavioural or psychological correlates. These findings should therefore be interpreted cautiously, but they are still compatible with the broader literature showing that substance-related behaviours and sleep interact in complex, often bidirectional ways.

Movement behaviours showed more mixed and outcome-specific associations. Greater sitting time was associated with longer sleep latency, which is consistent with the increasingly recognized links between sedentary lifestyles, poorer health profiles, and less favourable sleep patterns. Emerging evidence from 24 h movement behaviour frameworks as well as more broader frameworks suggests that excessive sedentary time is independently associated with adverse health and sleep outcomes, even among individuals who meet physical activity recommendations (Owen et al., 2010; Biswas et al., 2015; Ekelund et al., 2016; Kracht et al., 2024). By contrast, physical activity was positively associated with sleep latency and negatively associated with sleep efficiency and sleep quality in the final models, which differs from the dominant pattern in the broader literature. Current evidence generally supports a bidirectional relationship in which regular physical activity benefits sleep, particularly subjective sleep quality, while poor sleep may in turn reduce activity engagement (Lastella et al., 2024). Meta-analytic and umbrella review evidence further indicates that adherence to balanced 24 h movement behaviours, including adequate physical activity, reduced sedentary time, and sufficient sleep, is associated with more favourable overall health profiles (Kracht et al., 2024). The present inverse pattern may therefore reflect the specific characteristics of this multinational sample, the cross-sectional design, or measurement-related factors such as activity timing, occupational exertion, reverse causality, or residual confounding. Importantly, physical activity was not retained in the final insomnia model, suggesting that its relationship with sleep in the present sample may be more nuanced and more outcome-specific than commonly assumed. This is also coherent with the most recent analysis within MEDIET4ALL project, where movement behaviours showed distinct and sometimes counterintuitive associations depending on whether physical activity or sitting time was modelled as the primary outcome.

### 4.4. Strengths and Limitations

This study has several strengths. First, it is based on a large multinational sample of 4010 adults from ten Mediterranean and neighbouring countries, allowing sleep-related correlates to be examined across diverse sociocultural contexts. Second, the standardized multilingual survey design supported harmonized data collection across countries. Third, the study used established instruments and indices, including the Insomnia Severity Index, selected PSQI-derived sleep indicators, the MEDLIFE index, IPAQ-SF, SLSQ, DASS-21, SSPQ, and STuQL. Fourth, the analysis integrated sleep within the broader MEDIET4ALL lifestyle framework, allowing dietary, psychosocial, behavioural, and health-related correlates to be examined simultaneously within one standardized multinational dataset. This integrative design allowed the relative and outcome-specific patterns of these associations to be compared across insomnia severity and secondary sleep dimensions. Finally, the exploratory indirect association analyses provide a cautious hypothesis-generating extension of the primary regression findings and help contextualize the broader relationship between Mediterranean lifestyle-related factors, psychosocial well-being, and sleep outcomes.

Several limitations should also be acknowledged. First, the cross-sectional design precludes causal inference and does not allow temporal ordering of the observed associations. Second, all sleep outcomes and correlates were assessed by self-report, which may introduce recall bias, reporting bias, and measurement imprecision. Third, subjective sleep quality was assessed using an ordinal four-level response scale and treated as approximately continuous in the primary analyses to facilitate comparability across sleep outcomes. However, additional ordinal logistic regression sensitivity analyses yielded broadly comparable findings. Fourth, although the final model for insomnia severity demonstrated the strongest explanatory profile among the examined sleep outcomes, the explained variance for sleep duration, sleep latency, sleep efficiency, and subjective sleep quality remained comparatively modest, particularly for sleep duration. This suggests that additional biological, chronobiological, environmental, occupational, and contextual determinants influencing these secondary sleep dimensions were not comprehensively captured within the present survey. Accordingly, findings related to these secondary sleep outcomes should be interpreted more cautiously as exploratory correlational profiles rather than highly predictive explanatory models. In addition, the broad regional classification used in the present study may mask substantial sociocultural, environmental, and behavioural heterogeneity within regions. Therefore, the observed regional association should not be interpreted as reflecting a homogeneous ‘Mediterranean effect’, but rather as indicating the potential importance of broader contextual influences. The timing and context of data collection should also be considered. Data were collected during 2024 across countries characterized by varying social, economic, occupational, and geopolitical contexts, which may have contributed to unmeasured contextual heterogeneity influencing lifestyle, psychosocial, and sleep-related outcomes. Finally, the exploratory indirect association analyses were cross-sectional and hypothesis-generating in nature. Therefore, they cannot establish temporal ordering, causal mediation, or mechanistic pathways. The observed indirect associations may reflect bidirectional relationships, shared variance, or residual confounding among lifestyle-related, psychosocial, and sleep-related constructs. Longitudinal and intervention-based studies using repeated measures and objective sleep assessments are needed to clarify the temporal robustness of the proposed lifestyle–well-being–sleep framework.

### 4.5. Implications and Future Directions

The present findings have implications primarily for future research and for the broader conceptualization of sleep within multidimensional lifestyle-health frameworks.

First, the consistently stronger explanatory profile observed for insomnia severity suggests that insomnia-related symptoms may represent a particularly relevant sleep-health indicator within psychosocial and Mediterranean lifestyle research. By contrast, the comparatively lower explanatory capacity observed for sleep duration, sleep latency, sleep efficiency, and subjective sleep quality indicates that broader biological, chronobiological, occupational, environmental, and contextual determinants likely contribute importantly to these secondary dimensions of sleep health.

The present findings also support contemporary multidimensional conceptualizations of sleep health, in which different sleep dimensions are considered related but not interchangeable constructs (Buysse, 2014; Grandner, 2017). Future studies may therefore benefit from avoiding overly simplistic single-indicator approaches to sleep and instead examining how distinct dimensions of sleep health differentially relate to psychological well-being, distress, lifestyle behaviours, and social participation.

The consistent associations observed between psychological factors and multiple sleep outcomes highlight the importance of integrating mental well-being into sleep-health frameworks. However, given the cross-sectional nature of the present findings, these associations should be interpreted as correlational rather than causal. Nevertheless, the findings support the potential relevance of psychological well-being, stress regulation, emotional functioning, and life satisfaction within broader sleep-health promotion frameworks rather than focusing solely on behavioural sleep hygiene.

Similarly, the observed associations between Mediterranean dietary dimensions and sleep outcomes suggest that nutritional behaviours may represent a relevant, modifiable component of sleep health. Importantly, the distinction between dietary consumption patterns and dietary habits indicates that both the quality of food intake and the behavioural context of eating may play complementary roles in shaping sleep outcomes. This supports the need for more nuanced dietary recommendations that consider not only nutrient intake but also eating behaviours and daily routines. The independent association observed for region also suggests that future multinational sleep-health research should examine Mediterranean lifestyle not only through dietary composition, but also as a broader behavioural and sociocultural ecosystem. Broader contextual dimensions, including work–life organization, community participation, social rhythm regularity, and culturally embedded daily routines, may be relevant for understanding sleep-health profiles across countries.

Third, the role of social participation and its exploratory indirect association with selected sleep outcomes through life satisfaction points to the potential importance of social and environmental contexts within multidimensional sleep-health frameworks. These findings suggest that community-level and social participation-related factors may represent relevant components within broader sleep-health promotion strategies. However, intervention studies are required before concluding that modifying social participation, Mediterranean lifestyle behaviours, or psychosocial well-being directly improves sleep outcomes. From a research perspective, the cross-sectional nature of the present analyses underscores the need for longitudinal and experimental studies to clarify temporal relationships and potential causal pathways. Future studies should also incorporate objective sleep assessments (e.g., actigraphy or polysomnography) and explore additional determinants not captured in the present study, including occupational factors, environmental exposures, and broader sociocultural contexts. Recent global sleep-health perspectives increasingly emphasize that sleep duration, sleep quality, and sleep consistency are shaped by broader social, occupational, environmental, and cultural determinants beyond individual lifestyle behaviours alone (DelRosso, 2025).

Finally, given the comparatively modest explained variance observed for several secondary sleep outcomes, the present findings should not be interpreted as providing direct intervention targets on their own. Rather, they identify candidate correlational domains, particularly psychological well-being, distress, Mediterranean lifestyle-related dietary behaviours, and social participation, that warrant more focused examination in longitudinal, mechanistic, and intervention-based research.

## 5. Conclusions

In conclusion, the present study shows that insomnia severity and other sleep outcomes are associated with a multidimensional set of psychological, lifestyle, psychosocial, health-related, and sociodemographic factors within the MEDIET4ALL framework. Among the examined outcomes, insomnia severity demonstrated the strongest and most consistent explanatory profile, with psychological well-being and distress variables emerging as the dominant correlates. Mediterranean dietary dimensions and social participation showed comparatively smaller but statistically significant associations with selected sleep outcomes, including exploratory indirect associations involving life satisfaction.

Overall, the present findings support the view that sleep outcomes are embedded within a broader Mediterranean lifestyle and psychosocial framework involving psychological well-being, dietary behaviours, social participation, and contextual influences. The multinational design additionally suggests that broader sociocultural and regional characteristics may contribute to sleep-health profiles beyond Mediterranean dietary consumption patterns alone. Nevertheless, because of the cross-sectional design and the modest explanatory capacity observed for several secondary sleep outcomes, the findings should be interpreted cautiously as correlational and hypothesis-generating rather than causal or strongly predictive.

## Figures and Tables

**Table 1:** Final fully adjusted parsimonious regression model examining correlates of insomnia severity.

Variable	M7 B (95% CI)	M7 β	M7 *p*
Age	−0.028 (−0.039; −0.017)	−0.074	<0.001
Sex	0.619 (0.298; 0.94)	0.052	<0.001
Region	−0.761 (−1.09; −0.431)	−0.062	<0.001
BMI	0.036 (0.003; 0.07)	0.03	0.034
Smoking	−0.243 (−0.435; −0.051)	−0.033	0.013
Alcohol			
Health status	−0.913 (−1.187; −0.639)	−0.094	<0.001
MEDLIFE dietary consumption patterns	−0.113 (−0.192; −0.034)	−0.038	0.005
SLSQ total score	−0.14 (−0.177; −0.103)	−0.109	<0.001
DASS depression	0.17 (0.114; 0.226)	0.146	<0.001
DASS anxiety	0.208 (0.154; 0.262)	0.169	<0.001
DASS stress	0.199 (0.142; 0.256)	0.163	<0.001
No. of variables	11		
R^2^	0.294		
Adjusted R^2^	0.292		
ΔR^2^	0.294		
F change	151.49		

*p*-values lower than 0.001 are reported as *p* < 0.001. B = unstandardized regression coefficient; CI = confidence interval; β = standardized beta coefficient; Adjusted R^2^ = adjusted R-squared/adjusted coefficient of determination; F = F-statistic for the overall regression model; *p* for model = overall model *p*-value; SLSQ= Short Life Satisfaction Questionnaire.

**Table 2:** Final fully adjusted parsimonious regression models examining correlates of secondary exploratory sleep outcomes.

Variable	Sleep Duration B (95% CI)	β	*p*	Sleep Latency B (95% CI)	β	*p*	Sleep Efficiency B (95% CI)	β	*p*	Sleep Quality B (95% CI)	β	*p*
Age	−0.011 (−0.015; −0.007)	−0.099	<0.001									
Sex				4.313 (2.218; 6.407)	0.063	<0.001	−0.856 (−1.297; −0.415)	−0.059	<0.001	−0.047 (−0.095; 0.001)	−0.03	0.054
Region	−0.171 (−0.281; −0.062)	−0.048	0.002				−0.391 (−0.839; 0.057)	−0.026	0.087	−0.102 (−0.151; −0.053)	−0.062	<0.001
Employment	0.096 (0.053; 0.14)	0.068	<0.001	1.499 (0.66; 2.338)	0.053	<0.001	−0.254 (−0.432; −0.076)	−0.042	0.005			
Smoking	0.122 (0.057; 0.188)	0.058	<0.001	1.38 (0.115; 2.645)	0.033	0.033						
Alcohol				2.142 (0.399; 3.886)	0.038	0.016						
Health status	0.152 (0.06; 0.243)	0.054	0.001				0.514 (0.155; 0.872)	0.043	0.005	0.138 (0.099; 0.177)	0.106	<0.001
MEDLIFE dietary consumption patterns				−0.582 (−1.104; −0.059)	−0.034	0.029				0.016 (0.003; 0.028)	0.038	0.012
MEDLIFE dietary habits				−0.724 (−1.381; −0.066)	−0.033	0.031				0.016 (0; 0.031)	0.031	0.045
IPAQ score				0.001 (0; 0.001)	0.049	0.001	0 (0; 0)	−0.047	0.002	−0.00001561 (0; 0)	−0.043	0.005
Sitting time				0.398 (0.072; 0.724)	0.036	0.017						
SLSQ total score				−0.811 (−1.038; −0.583)	−0.109	<0.001	0.191 (0.143; 0.24)	0.121	<0.001	0.03 (0.024; 0.035)	0.171	<0.001
Anxiety	0.041 (0.024; 0.058)	0.114	<0.001	1.708 (1.486; 1.93)	0.24	<0.001	−0.338 (−0.384; −0.291)	−0.223	<0.001			
Stress	−0.052 (−0.069; −0.036)	−0.147	<0.001							−0.027 (−0.032; −0.021)	−0.162	<0.001
SSPQ total score	0.01 (0.005; 0.015)	0.059	<0.001							0.006 (0.004; 0.009)	0.082	<0.001
Constant												
No. of variables	8			10			7			9		
Adjusted R^2^	0.046			0.104			0.09			0.117		
*p* for model	0.048			0.106			0.092			0.119		
F	24.992			47.613			57.768			60.042		

*p*-values lower than 0.001 are reported as *p* < 0.001. B = unstandardized regression coefficient; CI = confidence interval; β = standardized beta coefficient; Adjusted R^2^ = adjusted R-squared/adjusted coefficient of determination; F = F-statistic for the overall regression model; *p* for model = overall model *p*-value; IPAQ score = International Physical Activity Questionnaire score; SSPQ = Short Social Participation Questionnaire; SLSQ = Short Life Satisfaction Questionnaire.

## Data Availability

The datasets generated and analyzed during the current study are not publicly available at this time as further analyses are ongoing, and additional publications based on these data are in preparation. Data may be made available upon reasonable request to the corresponding author once all planned analyses and publications are completed.

## Supplementary Materials

The following supporting information can be downloaded at: https://www.mdpi.com/article/10.3390/ejihpe16070096/s1, Table S1: Spearman correlations among sleep outcomes; Table S2: Hierarchical regression models examining insomnia severity across sequential conceptual domains; Table S3: Collinearity diagnostics for predictors included in the fully adjusted ISI model; Table S4: Sensitivity interaction analysis examining whether the association between Mediterranean dietary consumption patterns and insomnia severity differs according to region; Table S5: Ordinal logistic regression sensitivity analysis for subjective sleep quality; Table S6: Bootstrapped exploratory statistical indirect association analyses involving life satisfaction.
