# Supplementary material for: Sleep Characteristics and Insomnia Severity in Relation to Mediterranean Lifestyle Adherence and Psychosocial Wellbeing: Findings from the MEDIET4ALL International Survey
Source: Eur J Investig Health Psychol Educ. 2026 Jul 2;16(7):96. doi: 10.3390/ejihpe16070096 (PMC13409072; doi:10.3390/ejihpe16070096)
Supplement: Supplementary file 1 [file ejihpe-16-00096-s001.zip › ejihpe-4274860-supplementary.pdf]

Supplementary Table S1: Spearman correlations among sleep outcomes

Variable	1	2	3	4	5
1. ISI total score	1.000	−0.228 (<0.001)	0.422 (<0.001)	−0.450 (<0.001)	−0.546 (<0.001)
2. Sleep duration	−0.228 (<0.001)	1.000	−0.087 (<0.001)	0.277 (<0.001)	0.304 (<0.001)
3. Sleep latency	0.422 (<0.001)	−0.087 (<0.001)	1.000	−0.976 (<0.001)	−0.330 (<0.001)
4. Sleep efficiency	−0.450 (<0.001)	0.277 (<0.001)	−0.976 (<0.001)	1.000	0.374 (<0.001)
5. Subjective sleep quality	−0.546 (<0.001)	0.304 (<0.001)	−0.330 (<0.001)	0.374 (<0.001)	1.000

Note: Values are Spearman's rho with p-values in parentheses. Higher ISI scores indicate greater insomnia severity. Higher subjective sleep quality scores indicate better perceived sleep quality. The very strong inverse correlation between sleep latency and sleep efficiency likely reflects the mathematical interdependence of these parameters.

Supplementary Table S2. Hierarchical regression models examining insomnia severity across sequential conceptual domains

Predictor	M1 β	M2 β	M3 β	M4 β	M5 β	M6 β
Age	-0.16	-0.20	-0.19	-0.19	-0.09	-0.08
Sex	0.07	0.10	0.11	0.10	0.06	0.06
Region	-0.05	-0.04	-0.04	-0.04	-0.06	-0.06
Education	-0.06	-0.04	-0.03	-0.03	0.00	-0.01
Employment	0.03	0.02	0.02	0.02	0.02	0.02
Marital status	0.05	0.02	0.02	0.02	0.03	0.03
Living environment	0.02	0.01	0.01	0.01	0.00	0.00
BMI		0.06	0.05	0.05	0.03	0.03
Smoking		-0.05	-0.04	-0.04	-0.03	-0.03
Alcohol		0.11	0.11	0.11	0.02	0.02
Health status		-0.18	-0.18	-0.18	-0.09	-0.09
MEDLIFE dietary consumption patterns			-0.05	-0.04	-0.03	-0.04
MEDLIFE dietary habits			-0.05	-0.05	-0.02	-0.02
IPAQ score				-0.02	0.01	0.01
Sitting time				0.03	0.01	0.01
SLSQ total score					-0.11	-0.11
DASS depression					0.15	0.15
DASS anxiety					0.16	0.16
DASS stress					0.16	0.16
SSPQ total score						0.03
STuQL total score						0.00
N	7	11	13	15	19	21
R^2	0.034	0.087	0.093	0.094	0.296	0.297
Adjusted R^2	0.033	0.085	0.09	0.091	0.293	0.293
ΔR^2	0.034	0.053	0.006	0.001	0.202	0.001
F change	20.301	58.274	12.279	2.772	285.934	1.67

B = unstandardized regression coefficient; CI = confidence interval; β = standardized beta coefficient; Adjusted R^2 = adjusted R-squared / adjusted coefficient of determination; F = F-statistic for the overall regression model; p for model = overall model p-value; IPAQ score = International Physical Activity Questionnaire score; ISI = Insomnia Severity Index; SSPQ = Short Social Participation Questionnaire.

Supplementary Table S3. Collinearity diagnostics for predictors included in the fully adjusted ISI model

Predictor	Tolerance	VIF
Age	,531	1,884
Sex	,861	1,161
Region	,921	1,086
Education	,895	1,117
Employment	,919	1,089
Marital status	,646	1,548
Living environment	,940	1,064
BMI	,854	1,171
Smoking	,928	1,077
Alcohol	,868	1,152
Health status	,836	1,196
MEDLIFE dietary consumption patterns	,927	1,079
MEDLIFE dietary habits	,893	1,120
IPAQ score	,933	1,072
Sitting time	,970	1,030
SLSQ total score	,769	1,300
DASS depression	,288	3,468
DASS anxiety	,340	2,939
DASS stress	,305	3,282
SSPQ total score	,792	1,263
STuQL total score	,990	1,010

VIF: variance inflation factor. Tolerance = 1/VIF. Collinearity diagnostics were derived from the fully adjusted regression model (Model 6).

Supplementary Table S4. Sensitivity interaction analysis examining whether the association between Mediterranean dietary consumption patterns and insomnia severity differs according to region

Outcome variable: Insomnia Severity Index (ISI) total score

**Predictor**	**B**	**β**	**SE**	**t**	**p**	**95% CI LL**	**95% CI UL**
Region (Mediterranean vs non-Mediterranean)	0.712	0.058	0.172	4.132	<0.001	0.374	1.049
MEDLIFE dietary consumption patterns (centred)	−0.954	−0.081	0.206	−4.632	<0.001	−1.357	−0.550
Region × MEDLIFE dietary consumption patterns	0.672	0.034	0.329	2.044	0.041	0.027	1.316

Note: Model summary: $R^2 = 0.309$; adjusted $R^2 = 0.303$; $F(36, 3973) = 49.36$; $p < 0.001$; residual SE = 4.866. In the interaction model, region was coded as 0 = non-Mediterranean and 1 = Mediterranean. MEDLIFE dietary consumption patterns were mean-centred before computation of the interaction term

Supplementary Table S5. Ordinal logistic regression sensitivity analysis for subjective sleep quality

**Model component**	**Result**
Outcome	Subjective sleep quality, ordered from "very bad" to "very good" (1 to 4) perceived sleep quality
Model fitting test	$\chi^2(35) = 568.73$, $p < 0.001$
Nagelkerke pseudo-R^2	0.147
Test of parallel lines / proportional odds assumption	$p < 0.001$
Interpretation of assumption test	Some deviation from the proportional odds assumption was observed; therefore, results were interpreted cautiously as sensitivity analyses
Predictors associated with better sleep quality	MEDLIFE score, OR = 1.05, $p < 0.001$; life satisfaction, OR = 1.08, $p < 0.001$; social participation, OR = 1.01, $p < 0.001$; better health status, OR = 1.74, $p < 0.001$
Predictors associated with poorer sleep quality	Non-Mediterranean region, OR = 0.75, $p < 0.001$; stress, OR = 0.92, $p < 0.001$

Note: OR = odds ratio. The ordinal logistic model was used as a sensitivity analysis because subjective sleep quality was measured on a four-level ordered scale. Results were broadly consistent with the primary linear regression model, but interpreted cautiously because the proportional odds assumption was statistically violated.

Supplementary Table S6. Bootstrapped exploratory statistical indirect association analyses involving life satisfaction.

Pathway	Direct β	Direct p	Indirect β	95% CI (indirect)
A: MEDLIFE dietary consumption patterns → SLSQ → ISI	-0.1160	0.0092	-0.0504	[-0.0740, -0.0276]
B: MEDLIFE dietary consumption patterns → SLSQ → sleep quality	0.0203	0.0010	0.0066	[0.0036, 0.0098]
C: MEDLIFE dietary habits → SLSQ → sleep quality	0.0259	0.0010	0.0153	[0.0112, 0.0195]
D: Social participation → SLSQ → sleep quality	0.0052	0.0001	0.0039	[0.0031, 0.0047]
